# Are we underutilizing bone marrow and cord blood? Review of their role and potential in the era of cellular therapies

**DOI:** 10.12688/f1000research.20605.1

**Published:** 2020-01-17

**Authors:** Elisabetta Xue, Filippo Milano

**Affiliations:** 1 Clinical Research Division, Fred Hutchinson Cancer Research Center, 1100 Fairview Avenue N, Seattle, WA, 98109, USA; 2 Hematology and Bone Marrow Transplant Unit, San Raffaele Scientific Institute IRCCS, Milan, Italy

**Keywords:** Cord Blood, Bone Marrow, Hematopoietic Stem Cell Transplant, Immunotherapy, Regenerative Medicine

## Abstract

Since the first hematopoietic stem cell transplant, over a million transplants have been performed worldwide. In the last decade, the transplant field has witnessed a progressive decline in bone marrow and cord blood utilization and a parallel increase in peripheral blood as a source of stem cells. Herein, we review the use of bone marrow and cord blood in the hematopoietic stem cell transplant setting, and we describe the recent advances made in different medical fields using cells derived from cord blood and bone marrow.

## Introduction

**Figure 1.**
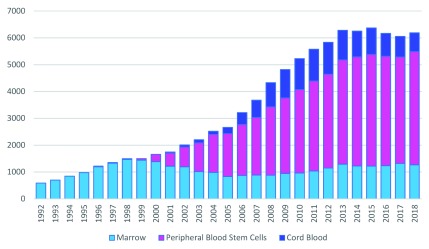
Transplants by cell source from 1992 to 2018, unrelated donor transplants. In the last decades, the number of hematopoietic stem cell transplants has progressively increased along with an expansion of peripheral blood as a source of stem cells. Data are reused from the National Marrow Donor Program (NMDP)/Be The Match with permission.

The role of bone marrow (BM) as a source of hematopoietic stem cells (HSCs) has been well established since 1868, when Neumann and Bizzozero used BM to reconstitute the hematopoietic system of rabbits. However, it took almost a century (1957) to perform the first allogeneic BM transplant in humans ^1^. A few decades later, two other sources of HSCs were successfully used in the transplant setting: in 1981, mobilized peripheral blood (PB) was adopted for an autologous transplant in a patient with chronic myelogenous leukemia ^2^, and in 1988, cord blood (CB) cells were transplanted in a patient with Fanconi’s anemia ^3^. Since the first BM transplant, over a million HSC transplants (HSCTs) have been performed ^4^. The widening of clinical indications, the gradual extension of eligibility criteria, and the inclusion of older patients have led to a constant increase in the numbers of HSCTs performed. However, in the last decade, PB has gradually become the most used source for HSCT because of (1) its ease of collection, (2) donors are spared from general anesthesia, and (3) the faster and higher engraftment rate associated with its use ( Figure 1), making it the first choice in more than 70% of adult allogeneic HSCTs and in almost all cases of autologous HSCTs ^5– 7^.

Despite being gradually confined to alternative hematopoietic graft sources, both CB and BM still retain unique biological and immunological properties and represent invaluable resources for the treatment of many medical conditions. In this review, we begin by addressing the pros and cons of CB and BM in the transplant setting. Next, we review their use in other fields such as immunotherapy and regenerative medicine.

## Cord blood

### Allogeneic transplantation

In recent decades, CB has emerged as a feasible alternative source of HSCs for pediatric and adult patients with hematological malignancies in need of an allogeneic transplant lacking a related or an unrelated donor (URD) ^8^. CB is a very attractive alternative source because of the increased level of HLA disparity that can be tolerated. This feature is of particular importance for patients from racial and ethnic minorities, for whom it can be difficult to find a URD ^9^. Indeed, in a recent National Marrow Donor Program study, an 8/8 HLA-matched URD was likely to be identified in 75% of white European patients, whereas a donor was identified in the URD registry in only around 20% and 35% of patients of African and Hispanic ancestry origin, respectively ^9^. Nowadays, CB has been used to transplant over 35,000 recipients and more than 730,000 CB units are stored and available worldwide in public banks ^10^.

Over the years, a number of retrospective studies have shown that CB transplantation (CBT) can yield disease-free survival (DFS) comparable to that of adult donor transplants in patients with hematologic malignancies ^11, 12^. In addition, many studies have confirmed low rates of malignant relapse after CBT compared with URD transplants, indicating that CB could be the preferred source for patients at high risk of relapse ^13^. CBT, when compared with the PB HSCT, also has the advantage of lower rates of chronic graft-versus-host disease (GvHD), which translates into lower long-term morbidity and mortality ^12^. The increased availability of CB units with a high cellular content, the use of double CB grafts, the direct intra-bone infusion of CB grafts to enhance the homing process, and numerous *ex vivo* expansion methods (major clinical trials using expanded CB units in HSCT are listed in Table 1) have further increased the potential application of this graft source ^14, 15^. Recently, Cohen *et al*. reported on outcomes of 22 patients with high- and very high-risk hematological malignancies who received a single CB unit expanded by using the UM171 technology ^16^. No graft failure was observed, and 1-year incidences of overall survival (OS), chronic GvHD-free DFS, and transplant-related mortality (TRM) were 90%, 74%, and 5%, respectively ^16^. Given that the high rate of early post-transplant morbidity and the requirement for intensive early post-transplant management have markedly slowed down the adoption of CBT, the low rate of TRM reported in the latter study is of high clinical interest. Targeted care strategies and development of feasible and safe CB expansion platforms can potentially increase the utilization of CBT.

**Table 1.** Summary of cord blood manipulation techniques in clinical trials.

Approach	Median CD34 ^+^ cell fold expansion (range)	Median infused (10 ^6^) CD34 ^+^/kg (range)	Median days to ANC engraftment (range)	Group
Expansion				
Cytokines (e.g., SCF, TPO, and G-CSF)	4 (0.1–20.0)	0.104 (0.0097–3.11)	28 days (15–49)	Shpall *et al*. ^27^
Copper-chelation	2.26 (0.67–19.2)	0.15 (0.05–4.63)	30 days (16–46)	De Lima *et al*. ^28^
Notch-ligand	164 (41–471)	6 (0.93–13)	16 days (7–34)	Delaney *et al*. ^29^
MSC co-culture	30.1 (0 – 137.8)	1.81 (0.09–9.88)	15 days (9–42)	De Lima *et al*. ^30^
Nicotinamide	72 (16–186)	3.5 (0.9–18.3)	13 days (7–26)	Horwitz *et al*. ^31^
SR-1	330 (67–848)	17.5 (1.4–48.3)	15 days (6–30)	Wagner *et al*. ^32^
UM171	28.1 (12.0–48.3)	28.75 (7.9–54.6)	18 days (12.5–20)	Cohen *et al*. ^16 ^
Homing				
CD26/DPP-4 inhibition	-	-	21 days (13–50)	Farag *et al*. ^33^
C3a priming	-	-	7 days (6–26)	Brunstein *et al*. ^34^
PGE2 exposure	-	-	17.5 days (14–31)	Cutler *et al*. ^35^
Fucosylation	-	-	17 days (12–34)	Popat *et al*. ^36^

ANC, absolute neutrophil count; C3a, complement fragment 3; DPP-4, dipeptidyl-peptidase IV; G-CSF, granulocyte colony-stimulating factor; MSC, mesenchymal stromal cell; PGE2, prostaglandin 2; SCF, stem cell factor; SR-1, StemRegenin-1; TPO, thrombopoietin.

### Immunotherapy

Unlike for HSCT, the use of CB cells for the development of adoptive therapies to treat post-transplant viral infections and malignant relapses has increased over the years. Virus-specific T (VST) cells are an appealing approach to prevent and treat viral reactivation in HSCT recipients, for whom long-duration antiviral treatments often cause unacceptable organ toxicity and virus resistance ^17^. Although CB T cells are virus-naïve ^18, 19^, Abraham *et al*. have recently reported on the successful generation and infusion of CB-derived VST cells directed against Epstein–Barr virus, adenovirus, and cytomegalovirus in CBT recipients as part of their antiviral prophylaxis or treatment or both ^20– 22^. The CB cells were obtained by separating an aliquot (20%) from the original CB graft, a process that did not delay or negatively impact neutrophil engraftment. Although the process was more time-consuming compared with VST cells generated from other sources, this approach was safe and feasible and showed efficacy in both preventing and treating end-organ viral infections ^22^

Among strategies to manage post-transplant malignant relapse, the infusion of donor-derived lymphocytes is often performed to boost the graft-versus-tumor effect, but for CBT recipients this option is not routinely available. Case reports of re-infusion of lymphocytes collected directly from the CBT recipients after immune reconstitution, with 23 or without 24 *ex vivo* expansion, have been described, as has infusion of T cells previously collected from the original CB grafts and subsequently expanded *ex vivo* ^25^. However, given the cost-effectiveness of these approaches, long-term safety and efficacy have to be carefully evaluated before they can enter the clinical routine.

Cellular immunotherapy, and more specifically autologous T cells genetically modified to express chimeric antigen receptor T (CAR-T) cells, has recently become the new frontier for the treatment for relapsed/refractory hematologic malignancies because of the ability to exert antitumoral cytotoxicity in an HLA-independent manner ^26^. The use of CAR-T cells has been explored mainly in the autologous setting, and very few studies have focused on the generation of allogeneic CAR-T cells. Given the naïve phenotype of CB-derived T cells as well as the large availability of CB units for the generation of cellular products along with the high *in vitro* proliferative capacity, CB represents a good and safe source of lymphocytes for the generation of allogeneic CAR-T cells. Indeed, several preclinical studies have investigated the use of CB for the generation of CB-derived T-cell lines possessing antileukemic activity by expressing CAR anti-CD19 ^37– 40^. Of note is the recent generation of CB-derived chimeric antigen receptor natural killer (CAR-NK) cells: unlike CAR-T cells, CAR-NK cells can also recognize target cells in a CAR-independent way, thus maintaining an antitumor effect in case of CAR-specific antigen downregulation on tumor cells ^41, 42^. To overcome the intrinsic short life span of CAR-NK cells, the incorporation of cytokine-encoding genes (for example, interleukin-15) has been successfully applied to this technology, allowing NK proliferation and survival ^41^. Third-party CAR-NK cells could be selected on the basis of killer-cell immunoglobulin-like receptor (KIR) mismatch between donor and recipient and used as an off-the-shelf product without risk of GvHD reaction; the goals would be to speed up the production and increase its feasibility. Clinical trials to determine the efficacy of CB-derived NK cells are ongoing.

Lastly, the use of CB-derived regulatory T (T-reg) cells is under investigation as part of prevention of GvHD. Brunstein *et al*. reported rates of acute and chronic GvHD of 9% and 0%, respectively, in 11 CBT recipients who received third-party CB T-reg cells on day +1 after transplant ^43^. In another study, five patients received an infusion of third-party CB-derived T-reg cells one day prior to PB SCT (n = 3) or double CBT (n = 2); the treatment was well tolerated, and four patients were off immune-suppression at the last follow-up evaluation ^44^. Further studies are needed to confirm these preliminary results.

### Regenerative medicine

The core of regenerative medicine is based on the identification of cells with repopulating and/or growth factor–secreting potential placed on biomimetic scaffolds forming a matrix for tissue regeneration. For this scope, CB cells are promising because of their (1) proliferative potential compared with adult-derived cells, (2) low immunogenicity, (3) low risk of transmitting infections of latent viruses, and (4) ease of collection ^45^.

With this in mind, different types of CB-derived cells have been examined for their “regenerative” capability. Particular attention has been given to the use of CB-derived mesenchymal stromal cells (MSCs) and endothelial progenitor cells (EPCs). CB-derived MSCs are pluripotent cells that display immune-modulatory properties and have the potential to differentiate into multiple lineages of mesodermal origin (mainly to produce osteoblasts, chondroblasts, and adipocytes) but also non-traditional lineages (for example, cardiomyocytes and hepatocytes). In addition, MSCs are known for the ability to accelerate healing processes in brain injury, in both *in vitro* and *in vivo* models, by inducing a neuroprotective anti-inflammatory microenvironment and promoting neurogenesis and revascularization. In animal models of dilated cardiomyopathy and myocardial ischemia, CB-derived MSCs have been used to improve the cardiac function by decreasing and preventing cardiac fibrosis, ventricle changes, and cellular apoptosis ^46, 47^. The use of CB-derived EPCs, like that of MSCs, has been extensively investigated in recent years; more specifically, endothelial colony-forming cells have shown the ability of homing into ischemic tissues, improving angiogenesis in preclinical models of ischemia ^48, 49^.

In the clinical setting, CB-based regenerative medicine has witnessed the greater innovations in the neurologic field, driven by the paucity of available treatments for progressive, non-reversible neurologic conditions. Sun *et al*. recently reported the results of a randomized placebo-controlled trial conducted in children affected by post-natal cerebral palsy; the authors reported an improvement in motor skill and white matter connectivity in patients who received a higher dose of autologous CB-derived total nucleated cells (TNCs) compared with patients who received either lower TNC dose or placebo ^50^. Similarly, Huang *et al*. reported a significant clinical improvement in 54 patients with cerebral palsy treated with allogeneic CB-derived MSCs ^51^. Encouraging results have also been achieved in autism spectrum disorders: repeated infusion of CB-derived MSCs were recently reported to be safe by Riordan *et al*. in a phase I trial, and behavioral improvement was reported in eight out of 15 evaluable patients ^52^. Furthermore, given the promising results in studies conducted on preclinical models ^53– 55^, the safety and therapeutic potential of CB-derived cells are under investigation in patients with neurodegenerative disorders, such as Parkinson’s disease or Alzheimer’s disease.

Ongoing phase I/II studies are exploring the role of CB-derived MSCs for the treatment of cardiac diseases. Of particular clinical interest is the observation that, in a randomized phase I/II clinical study, patients with heart failure who received an intravenous infusion of CB-derived MSCs had better post-ischemic myocardial remodeling and higher ventricular ejection fraction compared with the control group ^56^.

The safety and feasibility of CB-derived cells have been reported in several case series, but owing to the heterogeneity of these studies along with the lack of major comparative trials, their clinical efficacy still needs to be proven. Indeed, to date, none of these applications has been formally approved for clinical use.

## Bone marrow

### Allogeneic transplantation

**Figure 2.**
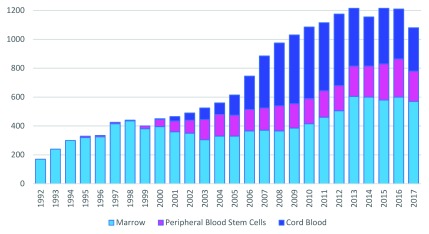
Transplants by cell source for pediatric patients from 1992 to 2017, unrelated donor transplants. In the pediatric population, defined as younger than 18 years, the most frequently used source is bone marrow. Data are reused from the National Marrow Donor Program (NMDP)/Be The Match with permission.

For decades, BM has been the preferred source for HSCT. In the early ’90s, several studies demonstrated a direct correlation between higher number of HSCs infused and a reduction of early transplant-related mortality ^57, 58^. This led to an increased use of granulocyte colony-stimulating factor (G-CSF)-primed PB, which in the last 15 years has gradually become the preferred graft source of HSCs ^5^. To date, aplastic anemia is the only disease for which the use of BM is mandatory because of the unacceptably high rate of chronic GvHD observed after PB HSCT ^59– 61^. BM also remains the preferred source of HSCs for pediatric patients ( Figure 2), for whom low cellularity is usually enough to ensure engraftment ^62^.

Many studies have compared BM and PB. In the first randomized clinical trial ever conducted in patients undergoing an HSCT with matched related donors, Bensinger *et al*. found a higher and faster rate of engraftment after PB, as compared with patients receiving BM, whereas no differences were observed for incidence of acute and chronic GvHD ^63^. The study suggested a better DFS in patients with advanced malignancies receiving PB ^63^. When focusing on long-term outcomes, Friedrichs *et al*. showed a higher incidence of chronic GvHD and longer need for immunosuppression therapy after HSCT using PB as source of HSCs ^64^. No differences were seen for OS and quality of life (for example, performance status and return to work) between PB and BM recipients ^64^. In 2012, the two graft sources were finally prospectively compared in the setting of URDs. The study showed a higher risk of graft failure but a lower rate of chronic GvHD among patients receiving BM; no significant differences were seen in OS between the two groups ^6^. The main finding of lower chronic GvHD led the authors to recommend the use of BM as the preferred source for HSCT, a recommendation that, however, has not been translated into clinical practice ^6^.

More recently, the use of BM has re-emerged in the setting of haploidentical transplants (that is, from half-matched related donors) that have been increasingly performed in the last decade ^65^. The original platform, described by Luznik *et al.*, using T cells-replete, haploidentical HSCT with post-transplant cyclophosphamide, included BM as the preferred HSC source ^66^; however, owing to the difficulty of obtaining BM, the same group explored the use of PB in the same setting, obtaining similar clinical results. As in URD transplants, these observations led to switching to PB as the preferred HSC source in the haploidentical setting as well ^66– 71^.

Although the use of BM is undoubtedly associated with a lower risk of chronic GvHD when compared with PB, the cumbersome process to obtain it and the slower time to engraftment have severely limited its application in the field of HSCT. To address the issue of slow engraftment and to reduce the rate of graft failure, some groups have investigated the use of a short course of G-CSF to stimulate BM before the harvest, documenting both an increase of progenitors and phenotypic changes in the lymphocyte component of the graft ^72, 73^. These observations led to the hypothesis that BM priming would have further lowered the rate of GvHD while improving engraftment. However, neither retrospective nor prospective studies comparing G-CSF–primed BM versus either unmanipulated BM or PB showed any differences in OS ^74– 77^. Moreover, given that this procedure would expose the donors both to a drug administration and to a BM harvest, the enthusiasm for this approach has quickly faded.

### Immunotherapy

The use of BM-derived MSCs has been widely investigated in the setting of severe, steroid-refractory GvHD. MSCs are capable of migrating into inflamed tissues affected by acute GvHD and actively inhibit T-cell proliferation, inducing a shift in the T cells toward a regulatory phenotype. Since the first report of successful use of MSCs in a case of refractory acute GvHD ^78^, several phase I/II studies have shown the safety and applicability of this approach. Although the heterogeneity of the studies using BM-derived MSCs has represented a major limitation for any definitive conclusions, the use of Remestemcel-L, an off-the-shelf BM-derived MSC product, has been approved for first-line treatment of acute GvHD and for the treatment of steroid-refractory acute GvHD. As first-line treatment, Remestemcel-L in combination with steroids led to 94% of overall responses, and 77% of those were complete ^79^. In the steroid-refractory GvHD setting, Remestemcel-L infusion, as single agent, led to a very promising 61% of overall response and significant improvement of survival ^80^. These results led to an open-label phase III trial that enrolled 55 children with steroid-refractory acute GvHD in 32 sites across the US, and 89% of patients had the most severe form (ClinicalTrials.gov Identifier: NCT02336230). The trial met the primary endpoint of day-28 overall response rate (69% versus 45% historical control rate) ^81^. Based on these results, the use Remestemcel-L is under evaluation by the US Food and Drug Administration as the first approved treatment of steroid-refractory acute GvHD.

### Regenerative medicine

BM cells are as valuable as CB cells as a source for the regenerative medicine field ^82^. Indeed, autologous and allogeneic BM aspirates, BM concentrates, and BM-derived cells have been extensively investigated to treat musculoskeletal conditions, especially for bone and cartilage damage such as osteoarthritis, bone fractures, or congenital skeletal malformations ^83, 84^.

Besides musculoskeletal conditions, BM-derived cells, such as EPCs, MSCs, and mononuclear cells, have been used to treat cardiac, endocrine (with a special focus on diabetes mellitus), and neurologic disorders. Whereas early phase I and II clinical trials have shown promising outcomes in patients receiving intracardiac injection of BM-derived cells after myocardial infarction, randomized placebo-controlled phase III studies have shown contradictory results in terms of overall clinical impact ^85– 87^.

BM-derived MSCs have been adopted for treating diabetes, showing the potential to improve the glycemic curve in preclinical models by differentiating *in vitro* into insulin-producing cells and by exerting a protective role against immune-mediated destruction of pancreatic beta cells in type 1 diabetes ^88, 89^. Within a cohort of 30 patients with type 2 diabetes requiring multiple oral anti-hyperglycemic drugs plus high-dose insulin, Bhansali *et al*. showed a significant reduction in insulin requirement after the infusion of BM-derived MSCs compared with patients receiving placebo ^90^.

More recently, several early phase clinical trials have investigated the role of BM-derived MSCs and mononuclear cells for the treatment of neurological conditions, including refractory multiple sclerosis, amyotrophic lateral sclerosis, traumatic brain injury, and cerebrovascular attacks ^91– 95^. In all cases, the treatment has been shown to be safe and feasible, although the clinical efficacy remains controversial.

Lastly, increasing interest has been raised around BM-derived MSC-secreted exosomes, extracellular nucleic acid-containing (for example, microRNA) and protein-containing vesicles that play a significant role in immune response and signal transduction. In preclinical studies, the administration of cell-free MSC-derived exosomes has been shown to improve cellular protection and regeneration in animal models of osteoporosis, bone fracture, optic nerve injury, traumatic brain injury, necrotizing enterocolitis, and other morbid conditions ^96– 100^.

## Conclusions

Much has been learned since the first HSCT performed in the late ’50s. The use of HSCT for patients with hematological disease continues to increase because of a constant decrease in transplant-related morbidity and mortality and a consequent improvement of clinical outcomes. Owing to the growing enthusiasm for PB as a source of stem cells, the use of CB and BM has decreased in recent years: according to the National Marrow Donor Program, CB and BM are being adopted in only 16% and 19% of all HSCTs, respectively. Although BM and CB transplantations are established practices for the treatment of hematological malignancies in adult and pediatric patients, the high transplant-related mortality due to delayed hematopoietic recovery (CB) and the difficulty of its acquisition (BM) have helped slow down the widespread adoption of both. There are several ongoing challenges to expand the use of CB. Although a number of methods to increase engraftment speed have been successfully investigated, the cost of CB grafts and the lack of substantial improvement in early post-transplant supportive care represent major unmet issues. For BM, the main limitation remains related to the difficulty and the invasiveness of its collection. Despite the undeniable advantage of a lower risk of chronic GvHD and consequently of a better quality of life, its use has not increased.

In recent years, we have learned more about the properties of CB and BM as well as their application to regenerative medicine and immunotherapy. Although there have not been major safety concerns regarding the use of CB-derived and BM-derived products, most of the clinical trials have been conducted on very small or heterogeneous cohorts (or both), using different cell populations, cell doses, and routes of administration. Randomized placebo-controlled studies are needed to better determine the efficacy of these approaches before translating them into standard clinical practice. With the development of new technologies allowing better characterization, selection, and expansion of different cell populations from CB and BM, we could envision a progressively higher utilization of these cell sources, not only in the transplant field but also in many other fields of medicine.

## Abbreviations

BM, bone marrow; CAR-NK cells, chimeric antigen receptor natural killer cells; CAR-T cells, chimeric antigen receptor T cells; CB, cord blood; CBT cord blood transplant; DFS, disease-free survival; EPC, endothelial progenitor cell; G-CSF, granulocyte colony-stimulating factor; GvHD, graft-versus-host disease; HSC, hematopoietic stem cell; HSCT, hematopoietic stem cell transplant; MSC, mesenchymal stromal cell; NK cells, natural killer cells; OS, overall survival; PB, peripheral blood; TNC, total nucleated cell; T-reg, regulatory T cell; TRM, transplant-related mortality; URD, unrelated donor; VST, virus-specific T cell.
